# Supplementary material for: A Likelihood Approach for Real-Time Calibration of Stochastic Compartmental Epidemic Models
Source: PLoS Comput Biol. 2017 Jan 17;13(1):e1005257. doi: 10.1371/journal.pcbi.1005257 (PMC5240920; doi:10.1371/journal.pcbi.1005257)
Supplement: S1 File — (TAR.GZ) [file pcbi.1005257.s014.tar.gz › HSPH_Online-SI-Revision/IPoi09/n1-extreme/n1-extreme_BENCH_table8.pdf]

1	R0	{2.95019, 14.2854}	{2.85477, 14.8514}	{{2.84915, 14.6465}, {2.86193, 15.0981}}	{3.23434, 3.96202}	inf	19.	36.8209	{10., 84.}	115.004	0.286307	newly	10.	14.5821	{3., 34.}	85.8209	0.298368	Reff	0.253716	0.232937	{0.173798, 0.298013}	17.3688	ar	924.	929.985	{915., 943.}	1.14525
2	R0	{2.67565, 16.946}	{2.81635, 19.898}	{{2.81635, 19.898}, {2.81635, 19.898}}	{5.25854, 17.42}	inf	31.	58.541	{28., 90.}	94.9762	0.235244	newly	14.	18.0164	{7., 29.}	57.4941	0.200109	Reff	0.232782	0.275356	{0.20841, 0.35486}	24.9412	ar	926.	924.623	{907., 943.}	1.19322
3	R0	{2.40066, 16.6543}	{2.81635, 19.898}	{{2.81635, 19.898}, {2.81635, 19.898}}	{17.3157, 19.4767}	inf	69.	90.5246	{48., 148.}	48.6814	0.157195	newly	26.	28.4918	{14., 45.}	43.7579	0.168828	Reff	0.249669	0.331452	{0.228124, 0.428085}	37.9992	ar	916.	924.623	{907., 943.}	1.50691
4	R0	{1.9294, 15.4361}	{2.67544, 19.9059}	{{2.64373, 19.7855}, {2.66225, 19.9254}}	{38.6668, 28.9566}	inf	64.	87.9048	{42., 145.}	57.7381	0.190641	newly	26.	27.8095	{13., 45.}	46.5201	0.199235	Reff	0.507432	0.349697	{0.230941, 0.487192}	37.7967	ar	796.	911.587	{895., 936.}	14.521
5	R0	{2.92859, 19.5393}	{1.30222, 1.35944}	{{1.30222, 1.35944}, {1.30222, 1.35944}}	{55.5342, 93.0425}	inf	46.	0.	{0., 0.}	100.	2.66276	newly	14.	0.	{0., 0.}	100.	2.14613	Reff	Refftrue	Reffmean	{0.5, 0.5}	200.	ar	927.	1.	{1., 1.}	99.8921
6	R0	{1.79622, 12.3148}	{2.60743, 19.6491}	{{2.50427, 19.1373}, {2.66225, 19.9254}}	{45.1618, 59.5564}	inf	28.	62.7015	{26., 120.}	128.092	0.28485	newly	16.	20.6716	{7., 40.}	66.5112	0.233857	Reff	0.555032	0.344347	{0.234278, 0.438247}	43.7154	ar	734.	900.493	{874., 920.}	22.6829
7	R0	{2.45855, 17.1776}	{2.97646, 18.6253}	{{2.96797, 18.4042}, {2.99088, 18.9406}}	{21.0655, 8.42776}	inf	47.	49.274	{21., 86.}	46.2256	0.196084	newly	18.	16.137	{6., 35.}	47.4886	0.233919	Reff	0.358948	0.225517	{0.172247, 0.281957}	37.65	ar	871.	937.356	{927., 949.}	7.61839
8	R0	{2.20009, 15.519}	{2.42205, 19.4475}	{{2.42205, 19.4475}, {2.42205, 19.4475}}	{10.0887, 25.3141}	inf	24.	36.1695	{11., 68.}	88.5593	0.270968	newly	12.	11.1356	{3., 24.}	69.6328	0.371311	Reff	0.378415	0.347215	{0.247049, 0.452923}	21.5214	ar	839.	879.458	{845., 908.}	4.94737
9	R0	{2.06493, 9.6414}	{2.84567, 15.1275}	{{2.84915, 15.0981}, {2.84915, 15.0981}}	{37.8095, 56.9015}	inf	11.	27.1905	{9., 54.}	154.69	0.326438	newly	7.	10.7143	{3., 23.}	87.9819	0.289778	Reff	0.489388	0.226131	{0.173798, 0.275732}	53.7932	ar	777.	927.889	{911., 946.}	19.4194
10	R0	{2.60775, 19.6936}	{2.41764, 19.4446}	{{2.42205, 19.4475}, {2.42205, 19.4475}}	{7.29012, 1.26421}	inf	57.	45.1404	{14., 88.}	59.4029	0.340109	newly	14.	15.1404	{3., 31.}	69.5489	0.306247	Reff	0.341615	0.365992	{0.254315, 0.465034}	23.2986	ar	889.	878.579	{845., 905.}	2.06816
11	R0	{1.99168, 8.13555}	{2.83659, 16.3305}	{{2.78517, 15.7559}, {2.8939, 16.8013}}	{42.4222, 100.731}	inf	7.	29.9452	{11., 58.}	328.571	0.564181	newly	6.	10.9452	{4., 19.}	99.3151	0.284995	Reff	0.478003	0.232304	{0.184838, 0.291489}	51.4011	ar	772.	926.315	{907., 943.}	19.989
12	R0	{1.79304, 9.18941}	{2.8, 15.9827}	{{2.78517, 15.7559}, {2.83341, 16.5525}}	{56.1592, 73.9255}	inf	15.	52.3194	{23., 96.}	248.981	0.472492	newly	9.	19.3472	{7., 36.}	124.846	0.301772	Reff	0.505637	0.25649	{0.200532, 0.328676}	49.7551	ar	725.	925.389	{907., 940.}	27.6398
13	R0	{1.84016, 18.3431}	{2.2499, 19.4095}	{{2.1968, 19.3849}, {2.26161, 19.3849}}	{22.2668, 5.81377}	inf	80.	53.6923	{19., 108.}	53.	0.33491	newly	33.	19.3846	{6., 39.}	57.1562	0.412494	Reff	0.651417	0.423392	{0.309841, 0.525035}	39.3084	ar	762.	852.215	{821., 876.}	11.8393
14	R0	{1.94221, 9.85971}	{2.98524, 18.7979}	{{2.96797, 18.4042}, {2.99088, 18.9406}}	{53.7034, 90.6532}	inf	4.	31.8514	{13., 58.}	696.959	0.819242	newly	2.	10.4459	{3., 19.}	425.	0.636446	Reff	0.388442	0.213339	{0.163338, 0.269179}	45.2872	ar	805.	937.189	{923., 950.}	16.421
15	R0	{1.78578, 12.0541}	{2.26161, 19.3849}	{{2.26161, 19.3849}, {2.26161, 19.3849}}	{26.6455, 60.8158}	inf	20.	56.0893	{21., 124.}	184.554	0.379414	newly	12.	18.7679	{6., 44.}	80.8036	0.235553	Reff	0.494661	0.408382	{0.298533, 0.53374}	26.3777	ar	732.	860.304	{840., 884.}	17.5278
16	R0	{1.94463, 15.6503}	{2.95125, 18.107}	{{2.83341, 16.5525}, {2.98558, 18.4749}}	{51.7641, 15.6975}	inf	49.	32.9571	{13., 55.}	46.7347	0.295136	newly	20.	11.4286	{4., 18.}	51.5714	0.353642	Reff	0.536718	0.217063	{0.169174, 0.277659}	59.5574	ar	757.	934.529	{916., 949.}	23.4516
17	R0	{2.08901, 16.4314}	{2.59711, 19.5978}	{{2.50427, 19.1373}, {2.66225, 19.9254}}	{24.3223, 19.2706}	inf	74.	75.1935	{35., 129.}	46.0331	0.197418	newly	26.	25.1774	{11., 46.}	46.464	0.210841	Reff	0.48465	0.359126	{0.247589, 0.471218}	35.2739	ar	826.	900.806	{874., 920.}	9.05647
18	R0	{2.76419, 18.56}	{2.98736, 18.8933}	{{2.96978, 18.8857}, {2.99088, 18.9406}}	{8.07358, 1.93009}	inf	52.	42.4211	{18., 82.}	43.4211	0.235868	newly	13.	13.3421	{4., 25.}	50.8097	0.233604	Reff	0.243249	0.220473	{0.164498, 0.27217}	17.0531	ar	920.	937.197	{924., 950.}	2.06093
19	R0	{2.3821, 17.1399}	{2.97795, 18.6326}	{{2.96797, 18.4042}, {2.99088, 18.9406}}	{25.0138, 8.70904}	inf	44.	47.8919	{21., 85.}	46.4373	0.185917	newly	11.	15.6486	{6., 31.}	65.602	0.205342	Reff	0.32873	0.226299	{0.172247, 0.287124}	32.3716	ar	883.	936.568	{921., 949.}	6.06654
20	R0	{2.78314, 19.6127}	{2.49687, 19.1652}	{{2.50427, 19.1373}, {2.50427, 19.1373}}	{10.2859, 2.28159}	inf	20.	41.3387	{15., 87.}	117.339	0.277062	newly	5.	13.0645	{4., 24.}	172.903	0.366916	Reff	0.18647	0.316196	{0.252931, 0.39317}	69.5691	ar	943.	891.903	{871., 909.}	5.41853
21	R0	{1.85124, 14.6794}	{2.42222, 19.4507}	{{2.42205, 19.4475}, {2.42205, 19.4475}}	{30.843, 32.5037}	inf	67.	93.1552	{39., 157.}	59.9331	0.201204	newly	31.	31.2586	{11., 58.}	46.5517	0.219189	Reff	0.596099	0.479617	{0.293068, 0.651531}	38.1858	ar	730.	879.017	{845., 905.}	20.4133
22	R0	{2.1028, 14.9852}	{2.95147, 18.815}	{{2.86501, 18.4249}, {2.99088, 18.9406}}	{40.3593, 25.557}	inf	31.	23.3467	{8., 43.}	47.7419	0.278434	newly	11.	7.93333	{2., 16.}	50.9091	0.31444	Reff	0.395326	0.211359	{0.166308, 0.263197}	46.5357	ar	840.	934.36	{919., 949.}	11.2333
23	R0	{1.86601, 10.2157}	{2.97754, 18.6116}	{{2.96797, 18.1089}, {2.99088, 18.9406}}	{59.5674, 82.1867}	inf	19.	49.7639	{19., 98.}	164.401	0.363099	newly	9.	16.3611	{6., 35.}	95.0617	0.252537	Reff	0.546741	0.235526	{0.178875, 0.288069}	56.9218	ar	747.	934.681	{917., 946.}	25.1246
24	R0	{2.34032, 10.0992}	{2.86193, 14.6465}	{{2.86193, 14.6465}, {2.86193, 14.6465}}	{22.288, 45.0263}	inf	19.	29.5246	{11., 48.}	75.2373	0.221118	newly	7.	12.2951	{3., 22.}	103.279	0.297132	Reff	0.278498	0.227547	{0.17744, 0.274745}	21.1064	ar	887.	929.213	{915., 944.}	4.77018
25	R0	{1.91548, 15.3401}	{2.65808, 19.8979}	{{2.64373, 19.7855}, {2.66225, 19.9254}}	{38.7682, 29.7119}	inf	36.	78.8226	{35., 146.}	121.55	0.291885	newly	14.	25.2419	{11., 44.}	89.977	0.245158	Reff	0.599545	0.341091	{0.244927, 0.483803}	45.5202	ar	765.	908.5	{894., 930.}	18.7582
26	R0	{2.75283, 10.6721}	{2.80133, 16.0341}	{{2.78517, 15.7559}, {2.80057, 16.2348}}	{1.76184, 50.2434}	inf	11.	38.8714	{15., 75.}	254.935	0.477176	newly	5.	14.6571	{5., 24.}	196.	0.387503	Reff	0.225732	0.249253	{0.192177, 0.311939}	18.0345	ar	926.	922.943	{904., 939.}	1.21567
27	R0	{2.08367, 19.7849}	{2.42222, 19.4507}	{{2.42205, 19.4475}, {2.42205, 19.4475}}	{16.2477, 1.68892}	inf	70.	53.1207	{18., 103.}	54.4089	0.317383	newly	16.	17.069	{5., 33.}	62.2845	0.279019	Reff	0.43132	0.383611	{0.256737, 0.481988}	27.3867	ar	833.	879.017	{845., 905.}	5.60293
28	R0	{2.64334, 17.1765}	{2.98441, 18.7879}	{{2.96797, 18.4042}, {2.99088, 18.9406}}	{12.9029, 9.38159}	inf	50.	55.1711	{21., 114.}	49.3947	0.203046	newly	17.	18.1316	{7., 40.}	54.1796	0.230212	Reff	0.227327	0.241397	{0.178078, 0.320736}	20.0514	ar	934.	935.158	{917., 950.}	1.13265
29	R0	{2.87008, 13.3872}	{2.64613, 19.8117}	{{2.64373, 19.7855}, {2.66225, 19.9254}}	{7.80304, 47.9899}	inf	16.	62.1127	{26., 128.}	288.204	0.500724	newly	8.	20.0282	{6., 39.}	160.563	0.344381	Reff	0.172205	0.324081	{0.23694, 0.423298}	88.1952	ar	941.	907.324	{892., 926.}	3.57875
30	R0	{2.06105, 19.6275}	{2.4188, 19.454}	{{2.42205, 19.4475}, {2.42625, 19.5287}}	{17.3577, 0.884045}	inf	18.	21.5571	{6., 44.}	70.2381	0.279804	newly	6.	6.25714	{1., 11.}	71.9048	0.360788	Reff	0.548239	0.31957	{0.247478, 0.380262}	41.7098	ar	747.	878.829	{852., 901.}	17.6477
31	R0	{2.30764, 13.435}	{2.88035, 17.2229}	{{2.80057, 16.2348}, {2.98125, 18.4042}}	{24.818, 28.1946}	inf	14.	30.9559	{12., 56.}	130.777	0.308473	newly	4.	10.5441	{3., 19.}	176.838	0.397761	Reff	0.313839	0.217765	{0.178078, 0.284925}	30.7834	ar	877.	932.485	{910., 949.}	6.32672
32	R0	{2.30565, 11.9237}	{2.82539, 16.4415}	{{2.78517, 15.7559}, {2.96797, 18.4042}}	{22.5419, 37.8896}	inf	9.	45.6719	{20., 72.}	407.465	0.624348	newly	6.	16.4063	{6., 24.}	177.083	0.3645	Reff	0.341236	0.244103	{0.187638, 0.308063}	29.8675	ar	853.	926.469	{907., 942.}	8.61298
33	R0	{2.24031, 18.582}	{2.42205, 19.4475}	{{2.42205, 19.4475}, {2.42205, 19.4475}}	{8.11227, 4.65773}	inf	83.	65.	{24., 134.}	50.1123	0.284414	newly	29.	21.9661	{7., 42.}	53.0099	0.317888	Reff	0.396535	0.407356	{0.261581, 0.528007}	27.7	ar	864.	879.458	{845., 908.}	2.5973
34	R0	{2.33306, 15.9759}	{2.95933, 18.8377}	{{2.86501, 18.4249}, {2.99088, 18.9406}}	{26.8433, 17.913}	inf	34.	55.8182	{23., 106.}	77.9985	0.21681	newly	12.	18.1948	{7., 39.}	72.1861	0.216539	Reff	0.354625	0.239031	{0.178187, 0.289366}	34.4584	ar	854.	934.896	{920., 949.}	9.47261
35	R0	{2.48357, 18.3573}	{2.98069, 18.7388}	{{2.96797, 18.4042}, {2.99088, 18.9406}}	{20.0164, 2.07799}	inf	55.	53.8	{21., 106.}	47.7091	0.216489	newly	20.	17.44	{6., 40.}	50.	0.252274	Reff	0.442075	0.232022	{0.176462, 0.287124}	47.4702	ar	835.	936.787	{925., 948.}	12.19
36	R0	{2.59548, 19.9046}	{2.67575, 19.9201}	{{2.66225, 19.898}, {2.66225, 19.9254}}	{3.09263, 0.107955}	inf	36.	73.5556	{32., 131.}	108.113	0.261521	newly	9.	23.381	{11., 39.}	161.905	0.358574	Reff	0.399704	0.332796	{0.244927, 0.436609}	31.4779	ar	863.	910.619	{894., 933.}	5.59142
37	R0	{2.29641, 16.9868}	{2.18608, 19.6819}	{{2.12588, 19.2116}, {2.26161, 19.9579}}	{4.80459, 15.8659}	inf	41.	49.6727	{20., 91.}	58.847	0.220394	newly	12.	15.5818	{5., 34.}	69.2424	0.253472	Reff	0.314608	0.445503	{0.327323, 0.61071}	42.2273	ar	877.	837.218	{796., 872.}	4.55271
38	R0	{2.65743, 15.4569}	{2.81635, 19.898}	{{2.81635, 19.898}, {2.81635, 19.898}}	{5.98021, 28.7322}	inf	23.	46.0492	{20., 71.}	106.344	0.246493	newly	9.	15.2787	{6., 25.}	87.6138	0.23466	Reff	0.318892	0.259797	{0.205594, 0.318248}	24.7238	ar	897.	924.623	{907., 943.}	3.13431
39	R0	{2.67953, 14.5132}	{2.98203, 18.9053}	{{2.96978, 18.8857}, {2.99088, 18.9406}}	{11.2893, 30.2626}	inf	24.	44.25	{16., 83.}	97.5329	0.263609	newly	6.	13.9474	{5., 27.}	140.351	0.323891	Reff	0.310825	0.226107	{0.175217, 0.27619}	29.5672	ar	894.	935.763	{921., 949.}	4.70093
40	R0	{1.94594, 11.928}	{2.97685, 18.8755}	{{2.96978, 18.8857}, {2.99088, 18.9406}}	{52.9772, 58.2451}	inf	36.	34.1212	{13., 63.}	53.5354	0.251596	newly	15.	11.1667	{3., 21.}	57.6768	0.35532	Reff	0.447566	0.215545	{0.169277, 0.27322}	51.8407	ar	796.	935.909	{921., 948.}	17.5765
41	R0	{1.66297, 10.0047}	{2.26161, 19.3849}	{{2.26161, 19.3849}, {2.26161, 19.3849}}	{35.9982, 93.7579}	inf	25.	56.0893	{21., 124.}	132.357	0.305218	newly	8.	18.7679	{6., 44.}	143.973	0.317869	Reff	0.53215	0.408382	{0.298533, 0.53374}	29.6667	ar	716.	860.304	{840., 884.}	20.1541
42	R0	{2.91568, 16.4453}	{2.85273, 14.9717}	{{2.84915, 14.6465}, {2.86193, 15.0981}}	{2.15907, 8.96091}	inf	25.	26.625	{8., 62.}	63.3889	0.279655	newly	14.	10.6944	{2., 25.}	59.127	0.408958	Reff	0.23617	0.226139	{0.171716, 0.280469}	14.3889	ar	919.	928.792	{914., 941.}	1.34355
43	R0	{2.55898, 15.8889}	{2.98432, 18.9102}	{{2.96978, 18.8857}, {2.99088, 18.9406}}	{16.6214, 19.0153}	inf	46.	76.3553	{35., 143.}	76.9737	0.219694	newly	20.	24.8947	{11., 46.}	52.1053	0.191988	Reff	0.30196	0.260236	{0.187096, 0.314042}	22.5225	ar	904.	935.829	{921., 949.}	3.58786
44	R0	{2.28649, 15.9017}	{2.82258, 19.9014}	{{2.81635, 19.898}, {2.81635, 19.898}}	{23.4461, 25.1527}	inf	33.	71.3387	{32., 105.}	118.426	0.280134	newly	11.	22.5484	{10., 36.}	111.144	0.260454	Reff	0.324682	0.295214	{0.214043, 0.380207}	26.0051	ar	880.	925.048	{907., 942.}	5.11913
45	R0	{2.65121, 18.1639}	{2.94878, 18.1093}	{{2.80057, 16.2348}, {2.98125, 18.4749}}	{11.229, 2.91429}	inf	63.	86.6494	{36., 169.}	59.1837	0.197849	newly	16.	28.7922	{12., 55.}	91.3149	0.251929	Reff	0.230655	0.270928	{0.184838, 0.367878}	26.2571	ar	928.	935.338	{914., 950.}	1.47363
46	R0	{2.52291, 18.0251}	{2.81635, 19.898}	{{2.81635, 19.898}, {2.81635, 19.898}}	{11.631, 10.3905}	inf	48.	46.0492	{20., 71.}	49.1462	0.215646	newly	15.	15.2787	{6., 25.}	53.6612	0.234707	Reff	0.32041	0.259797	{0.205594, 0.318248}	25.0028	ar	894.	924.623	{907., 943.}	3.45839
47	R0	{2.22618, 17.1225}	{2.81635, 19.898}	{{2.81635, 19.898}, {2.81635, 19.898}}	{26.5104, 16.2097}	inf	64.	90.5246	{48., 148.}	54.0984	0.163975	newly	23.	28.4918	{14., 45.}	48.6814	0.165771	Reff	0.454141	0.331452	{0.228124, 0.428085}	38.844	ar	850.	924.623	{907., 943.}	8.77917
48	R0	{2.04405, 16.073}	{2.81765, 19.8981}	{{2.81635, 19.898}, {2.81635, 19.898}}	{37.8463, 23.798}	inf	45.	72.2459	{32., 105.}	71.1111	0.195587	newly	18.	22.7869	{10., 36.}	55.7377	0.183521	Reff	0.533497	0.29864	{0.216859, 0.380207}	48.3344	ar	779.	924.525	{907., 942.}	18.6809
49	R0	{2.84396, 18.097}	{2.86193, 14.6465}	{{2.86193, 14.6465}, {2.86193, 14.6465}}	{0.631865, 19.0667}	inf	41.	19.8033	{8., 31.}	58.9764	0.416737	newly	16.	7.54098	{2., 13.}	59.6311	0.474794	Reff	0.170638	0.220181	{0.171716, 0.269021}	30.3397	ar	952.	929.213	{915., 944.}	2.40391
50	R0	{1.85341, 18.0975}	{2.97416, 18.8136}	{{2.96797, 18.4042}, {2.99088, 18.9406}}	{60.4696, 3.95667}	inf	40.	18.8923	{5., 35.}	62.3846	0.474494	newly	17.	6.12308	{2., 14.}	66.6968	0.57685	Reff	0.498567	0.200807	{0.163338, 0.246492}	59.7231	ar	754.	937.046	{922., 949.}	24.2767
